# Relationship Between Plasma Osteopontin and Arginine Pathway Metabolites in Patients With Overt Coronary Artery Disease

**DOI:** 10.3389/fphys.2020.00982

**Published:** 2020-08-06

**Authors:** Donato Moschetta, Matteo Nicola Dario Di Minno, Benedetta Porro, Gianluca L. Perrucci, Vincenza Valerio, Valentina Alfieri, Ilaria Massaiu, Alexander N. Orekhov, Alessandro Di Minno, Paola Songia, Viviana Cavalca, Veronika A. Myasoedova, Paolo Poggio

**Affiliations:** ^1^Unità per lo Studio delle Patologie Aortiche, Valvolari e Coronariche, Centro Cardiologico Monzino IRCCS, Milan, Italy; ^2^Dipartimento di Scienze Farmacologiche e Biomolecolari, Università degli Studi di Milano, Milan, Italy; ^3^Dipartimento di Scienze Mediche Traslazionali, Università degli Studi di Napoli Federico II, Naples, Italy; ^4^Unità di Metabolomica, Centro Cardiologico Monzino IRCCS, Milan, Italy; ^5^Unità di Medicina Rigenerativa e Biologia Vascolare, Centro Cardiologico Monzino IRCCS, Milan, Italy; ^6^Dipartimento di Medicina Clinica e Chirurgia, Università degli Studi di Napoli Federico II, Naples, Italy; ^7^Institute of General Pathology and Pathophysiology, Russian Academy of Medical Sciences, Moscow, Russia; ^8^Dipartimento di Farmacia, Università degli Studi di Napoli Federico II, Naples, Italy

**Keywords:** atherosclerosis, endothelial dysfunction, OPN, nitric oxide, citrulline

## Abstract

**Introduction:**

Osteopontin (OPN) is involved in ectopic calcification. Its circulating form is upregulated in coronary artery disease (CAD) patients. Circulating OPN levels positively correlate with oxidative stress, one of the major triggers of endothelial dysfunction. Endothelial dysfunction is, in turn, associated with reduced nitric oxide (NO) bioavailability due to the impaired arginine pathway. The aim of this study was to better understand the correlations between OPN, oxidative stress markers, and the arginine pathway metabolites.

**Methods and Results:**

ELISA and mass spectrometry techniques have been used to evaluate circulating OPN and arginine pathway/oxidative stress metabolites, respectively, in twenty-five control subjects and thirty-three patients with overt atherosclerosis. OPN positively correlates with 2,3-dinor-8isoPGF2a levels (*p* = 0.02), ornithine (*p* = 0.01), ADMA (*p* = 0.001), SDMA (*p* = 0.03), and citrulline (*p* = 0.008) levels only in CAD patients. In addition, citrulline positively correlated with ADMA (*p* = 0.02) levels, possibly as result of other sources of citrulline biosynthetic pathways.

**Conclusion:**

The association between OPN and impaired arginine/NO pathway could play a role in the inhibition of endothelial NO synthase (eNOS) and/or in the arginase activation in the context of CAD patients. However, further studies are needed to verify the cause-effect relationship between OPN, oxidative stress, and arginine/NO pathway dysregulation.

## Introduction

Osteopontin (OPN) is a phosphoglycoprotein secreted by different cellular types (monocytes, macrophages, cardiac fibroblasts, vascular smooth muscle cells, and endothelial cells), implicated in many molecular and cellular pathophysiological processes, including ectopic calcification (Cho and Kim, 2009). It has been shown that OPN plays an important role in the atherosclerotic plaque formation as well as in coronary artery diseases (CAD) (Wolak, 2014). In particular, several studies showed that circulating OPN levels are elevated in coronary artery disease (CAD) patients (Abdel-Azeez and Al-Zaky, 2010; Tousoulis et al., 2013; Wolak, 2014; Maniatis et al., 2019) and correlated with the disease extent and severity (Ohmori et al., 2003; Momiyama et al., 2010; Wolak, 2014). Indeed, circulating OPN has been proposed as a predictor of major cardiac events, such as acute myocardial infarction and ischemic heart disease (Georgiadou et al., 2010; Okyay et al., 2011). These observations, taken together with large literature evidences, corroborate the direct link between OPN and CAD development/progression (Wolak, 2014).

In addition, the upregulation of OPN transcription is also driven by oxidative stress (Branchetti et al., 2013) that represents one of the main initial atherosclerotic triggers, leading also to endothelial dysfunction (Incalza et al., 2018). Indeed, circulating OPN positively correlates with malondialdehyde levels, a recognized biomarker of oxidative stress (Cavalca et al., 2001; Georgiadou et al., 2008).

It has also been shown that increased levels of reactive oxygen species, in patients with CAD, lead to a progressive endothelial dysfunction (Incalza et al., 2018). Furthermore, it has been shown that endothelial vascular function impairment is associated with high OPN levels (Shemyakin et al., 2012; Schreier et al., 2016; Batko et al., 2019).

The endothelial dysfunction, among other causes, is associated with the impairment of the nitric oxide (NO) pathway, where the NO synthase (NOS) plays a pivotal role (Yang and Ming, 2013). NOS, using arginine as substrate, produces NO equimolarly to citrulline (Morris, 2007). Then, NO diffuses locally and mediates endothelium-dependent vasodilatation, acting on adhesion molecules and avoiding the infiltration of inflammatory cells and subsequent detrimental effects (Tousoulis et al., 2012). Undeniably, the reduction of NO bioavailability have a crucial importance in cardiovascular diseases (Cavalca et al., 2013; Eligini et al., 2013). Thus, in this study, we investigated the link between circulating OPN, oxidative stress, and endothelial dysfunction. We, therefore, performed an association study to explore the dysregulation of the arginine pathway and different oxidative stress markers in patients with overt CAD requiring surgical myocardial revascularization.

## Materials and Methods

### Study Population

Thirty-three patients that underwent coronary artery bypass grafting (CABG) and twenty-five control subjects were enrolled in the study between January and June 2011 at Centro Cardiologico Monzino IRCCS. Pre-operative inclusion criteria were isolated surgical myocardial revascularization, elective surgery, age more than 18 years old, ejection fraction >30% and normal sinus rhythm. Exclusion criteria were prior cardiac surgery, rheumatic heart disease, endocarditis, active malignancy, chronic liver, and kidney diseases, calcium regulation disorders (hyperparathyroidism, hyperthyroidism and hypothyroidism) and chronic or acute inflammatory states (sepsis, autoimmune disease and inflammatory bowel disease). The Institutional Review Board and Ethical Committee of Centro Cardiologico Monzino (IRCCS) approved the study. Written informed consent to participate in this prospective observational study was obtained from all enrolled patients. The study protocol was conformed to the ethical guidelines of the 1975 Declaration of Helsinki.

### Blood and Urine Sampling

Whole blood: 6 mL of peripheral blood sample was drawn from patients while fasting, into tubes containing EDTA (9.3 mM, Vacutainer Systems, Becton Dickinson, Franklin Lakes NJ, United States) kept on ice. 250 μL of whole blood was immediately precipitated with 250 μL of 10% trichloroacetic acid (Sigma-Aldrich, Darmstadt, Germany) plus 1 mM EDTA solution. Samples were stored at −80°C until analysis.

Plasma EDTA: anti-coagulated EDTA blood was centrifuged at 1700 g for 10 min at 4°C within 30 min after being drawn. Plasma was separated and aliquots were stored at −80°C until analysis.

Urine: urine collection was carried out the night before surgery or the night before the visit and samples stored at −80°C until analysis.

### Osteopontin Evaluation

Plasma levels of soluble OPN were measured with an enzyme-linked immunosorbent assay (ELISA) kit (Quantikine, R&D) following manufacturer instructions.

### Oxidative Stress Markers Measurement

Reduced (GSH) and oxidized glutathione (GSSG) forms were determined in whole blood by liquid chromatography-tandem mass spectrometry (LC-MS/MS) method (Squellerio et al., 2012; Valerio et al., 2019). The separation of analytes was conducted on a Luna PFP analytical column (100 mm × 2.0 mm, 3 m, Phenomenex) maintained at 35°C under isocratic conditions (flow rate of 250 μL/min, mobile phase:1% methanol in 0.75 mM ammonium formate adjusted to pH 3.5 with formic acid). LC-MS/MS analysis was performed using an Accela HPLC (high performance liquid chromatography) system coupled with a triple quadrupole mass spectrometer TSQ Quantum Access (Thermo Fisher Scientific, Waltham, MA, United States) equipped with an electrospray ionization (ESI) source working in multiple reaction monitoring (MRM) and in positive ionization mode.

Data were obtained after comparison with calibration curves using GSH and GSSG pure standard solutions (Sigma-Aldrich, Darmstadt, Germany). The intra- and inter-CVs (%) obtained with standard samples were <5% for both the analytes. The limits of detection were 0.031 μmol/L for GSH and 0.008 μmol/L for GSSG. Levels of GSH and GSSG were corrected for haemoglobin (Hb) and expressed as μmol/g Hb.

Urinary 2,3-dinor-8isoPGF2a was detected by LC-MS/MS method according to Cavalca et al. (2010). The urinary concentration was calculated from the area ratio of the ion peaks of the 2,3-dinor-8isoPGF2a over the deuterated standard (8-iso-PGF2a-d4). The estimated values were corrected for the urinary creatinine levels and expressed as pg/mg of creatinine.

### Arginine Pathway Analytes Measurement

The assessment of arginine, ornithine, citrulline, asymmetric dimethylarginines (ADMA), and symmetric dimethylarginine (SDMA) was performed by LC-MS/MS using a target metabolomic approach (Squellerio et al., 2011). Briefly, the chromatographic analysis was conducted on a Luna HILIC (hydrophilic interaction liquid chromatography) analytical column (50 mm × 2.0 mm, 3 μm, Phenomenex, Torrance, CA, United States). The mobile phases consisted of aqueous 1.5 mM ammonium formate (pH 3.2) (A) and 1.5 mM ammonium formate in acetonitrile/methanol (95.5:0.5, v/v) (pH 3.2) (B) at a flow rate of 250 μL/min. The mobile phase gradient ran from 10% A to 70% A over 7 min, from 70% A to 94.5% A over 2 min and was held at 94.5% A for 5 min, returning to 10% A over 2 min and held at 10% A for re-equilibration. The sample injection volume was 10 μL and the column temperature was set at 30°C. Total run time per sample, including column cleaning and re-equilibration, was 25 min. The mass spectrometric analysis was performed using a TSQ Quantum Access (Thermo Fisher Scientific, Waltham, MA, United States) triple quadrupole mass spectrometer equipped with ES) interface operating in MRM and positive ionization mode. The LOQ value is 0.25 M for all compounds, making this method suitable for the analysis of samples containing relatively low concentrations of the analytes, with a satisfactory precision as documented by the intra- and inter-day CVs of less than 10%. The method is linear in a wide range of concentrations (between 0 and 20 μM), with correlation coefficients greater than 0.99 and limit of detection (LOD) around 3–10 nm for all compounds. Global arginine bioavailability (GABR) was calculated as the ratio of arginine levels and the total amount of ornithine plus citrulline levels. GABR is an index of circulating arginine bioavailability associated with markers of endothelial dysfunction and increased risk of cardiovascular mortality (Morris et al., 2005; Sourij et al., 2011).

### Statistical Analyses

Continuous variables were analyzed using Student’s *T*-test and summarized as mean ± SD, while categorical ones were analyzed using Chi-square test and summarized as frequency (n) and percentage (%). Circulating biomarkers were analyzed by the Pearson product-moment correlation coefficient (*r*_*p*_) and plotted using Graphpad Prism v7.0. A value of *p* ≤ 0.05 was deemed statistically significant.

## Results

Demographic and clinical characteristics, as well as pharmacological therapies of the study population are listed in Supplementary Table S1. As previously reported by other authors (Tousoulis et al., 2013; Wolak, 2014; Maniatis et al., 2019), circulating OPN levels were lower in controls compared to the CAD patients (57.76 ± 9.8 vs 68.37 ± 24.2 pg/ml, respectively, *p* = 0.04, Supplementary Figure S1).

### Osteopontin and Oxidative Stress

We assess the possible relationship between OPN levels and oxidative stress status, represented by 2,3-dinor-8isoPGF2a and the ratio between the reduced (GSH) and the oxidized (GSSG) forms of glutathione, in patients before the surgical intervention.

**FIGURE 1:**
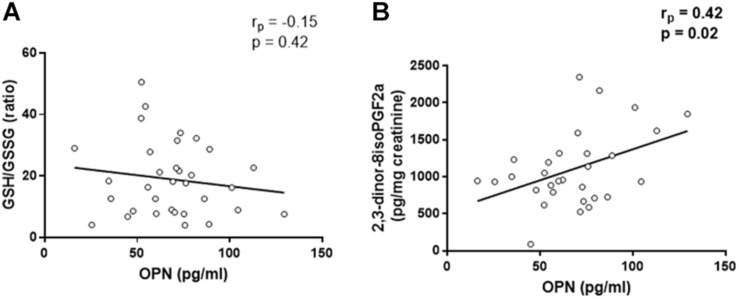
Linear regression analyses between osteopontin (OPN) and GSH/GSSG ratio **(A)** and 2,3-dinor-8isoPGF2a **(B)**.

Linear regression analysis reported that there was no significant association between OPN levels and GSH/GSSG ratio, in controls (*r*_*p*_ = 0.002, *p* = 0.99, Supplementary Figure S2A), as well as in CAD patients (*r*_*p*_ = −0.15, *p* = 0.42, Figure 1A). The same analysis showed that there was no association between OPN levels and 2,3-dinor-8isoPGF2a urine levels in control group (*r*_*p*_ = −0.38, *p* = 0.08, Supplementary Figure S2B). However, OPN levels were directly correlated with 2,3-dinor-8isoPGF2a urine levels in CAD patients (*r*_*p*_ = 0.42, *p* = 0.02, Figure 1B).

### Osteopontin and Arginine Pathway Metabolites

**FIGURE 2:**
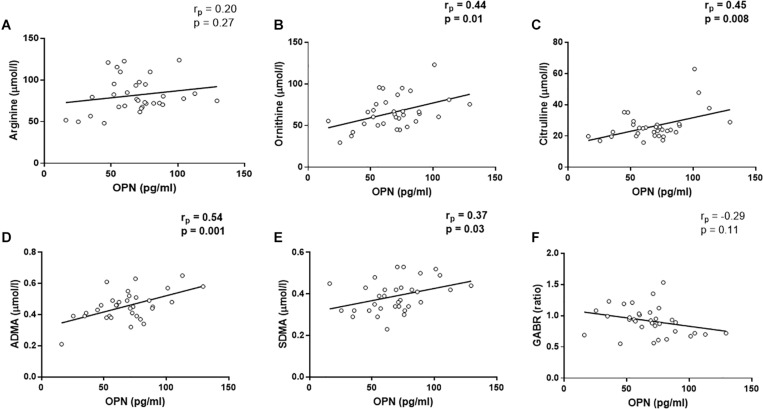
Linear regression analyses between osteopontin (OPN) and arginine **(A)**, ornithine **(B)**, Citrulline **(C)**, asymmetric dimetilarginine [ADMA, **(D)**], symmetric dimethilarginine [SDMA, **(E)**], and global arginine bioavailability [GABR, **(F)**].

Since arginine is the substrate of NOS, we evaluated the metabolites involved in arginine pathway as representative molecules of NO production (Morris, 2007, 2016). In the control group, there were no correlations between the considered metabolites and circulating OPN levels (arginine, *r*_*p*_ = −0.33, *p* = 0.11, ornithine, *r*_*p*_ = −0.04, *p* = 0.85, citrulline, *r*_*p*_ = 0.07, *p* = 0.75, ADMA, *r*_*p*_ = −0.30, *p* = 0.14, SDMA, *r*_*p*_ = −0.23, *p* = 0.28, GABR, *r*_*p*_ = 0.30, *p* = 0.14, Supplementary Figure S3). In CAD patients, the linear regressions showed that OPN levels were not associated with arginine levels (*r*_*p*_ = 0.20, *p* = 0.27, Figure 2A) and the global arginine bioavailability (GABR, *r*_*p*_ = −0.29, *p* = 0.11, Figure 2F). However, OPN levels were positively correlated with ornithine (*r*_*p*_ = 0.44, *p* = 0.01, Figure 2B), ADMA (*r*_*p*_ = 0.54, *p* = 0.001, Figure 2D), and SDMA (*r*_*p*_ = 0.37, *p* = 0.03, Figure 2E) levels.

**FIGURE 3:**
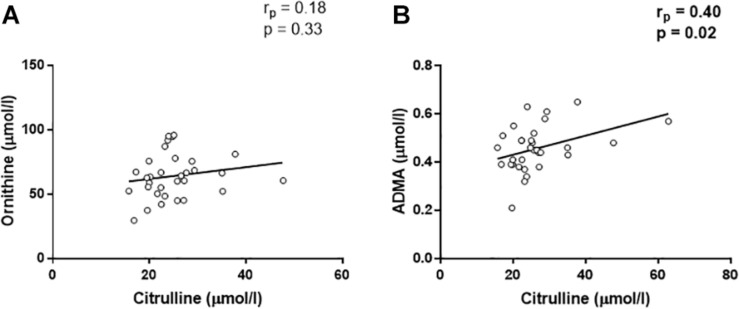
Linear regression analyses between citrulline and ornithine **(A)** and asymmetric dimetilarginine [ADMA, **(B)**].

In addition, a positive correlation was found between OPN and citrulline (*r*_*p*_ = 0.45, *p* = 0.008, Figure 2C). Citrulline is known to be produced by (i) NOS from arginine, equimolarly with NO, (ii) ornithinetranscarbamilase (OTC) form ornithine, and (iii) dimethylarginine dimethylaminohydrolase (DDAH) from ADMA. In this regard, citrulline was not associated with ornithine (*r*_*p*_ = 0.18, *p* = 0.33, Figure 3A), although, we found that citrulline levels were associated with ADMA (*r*_*p*_ = 0.40, *p* = 0.02, Figure 3B) levels.

## Discussion

To our knowledge, we show for the first time that OPN could be linked to the pathological dysregulation of the arginine pathway in CAD patients.

As largely reported before also CAD patients in our cohort showed high levels of circulating OPN. Nonetheless, CAD patients were characterized by an increased oxidative stress status and impaired endothelial function associated with a NO bioavailability reduction (Abdel-Azeez and Al-Zaky, 2010; Yang and Ming, 2013; Incalza et al., 2018). Recently some authors reported that high OPN levels could in some way interfere with vessel endothelial function (Shemyakin et al., 2012; Batko et al., 2019; Maniatis et al., 2019). – In this study, we investigated the relationship between OPN, oxidative stress, and endothelial dysfunction taking into account the arginine metabolism. In our cohort, plasma OPN levels correlated with urinary 2,3-dinor-8isoPGF2a, in agreement with literature evidences on the intensified production of OPN caused by an increased systemic oxidative stress status. However, we did not see any significant association between plasma OPN and GSH/GSSG ratio. These data suggest that lipid peroxidation may be the main process induced by the oxidative stress in the context of CAD, instead of protein oxidation. The link between OPN and lipid peroxidation is corroborated by the lack of any association between OPN and 2,3-dinor-8isoPGF2a in the control group.

It has been shown that OPN could interfere with the arginine pathway by inhibiting the inducible form of the NOS (iNOS) enzyme (Singh et al., 1995; Rabenstein et al., 2016). Thus, it is likely that the same mechanisms could cause an inhibition of endothelial NOS (eNOS) enzyme as a result of increased OPN levels. In this scenario, the reduction of NO synthesis, in combination with increased oxidative stress status, would favor the atherosclerotic milieu (Mahdi et al., 2019). For this purpose, we analyzed the metabolites of the arginine pathway both in controls and CAD patients. We found no correlation between OPN and any metabolite in control group, while in the in CAD patients, we found that OPN directly correlated with several metabolites belonging to the arginine pathway. Indeed, we found positive correlations between OPN, ADMA, SDMA, and ornithine. SDMA is not only an inhibitor of the arginine transporter CAT (Closs et al., 1997), but also a pro-inflammatory molecule (Chen et al., 2012), like OPN (Icer and Gezmen-Karadag, 2018). In the context of CAD, SDMA could play both roles acting in synergy with OPN in the development of the inflammation. However, we also observed positive correlations between citrulline and OPN. To explain this last correlation, we have to take into account that citrulline is normally produced equimolarly to NO from arginine by eNOS, but other sources of its production are known (Morris, 2007). In particular, citrulline could derive from ADMA by DDAH activity. Indeed, in our cohort we found a positive correlation between citrulline and ADMA, indicating that high levels of citrulline could be due to the activity of DDAH enzyme.

In 2012, Shemyakin et al. (2012) showed, in CAD patients, an improved endothelial functionality probably due to the inhibition of arginase. This evidence suggests that arginase activation reduces arginine bioavailability, thus NOS-mediated NO production, fundamental to maintain the endothelial function. Of notice, it has been reported a possible interaction between OPN and arginase (Partridge et al., 2008). Thus, it is likely that OPN could stimulate arginase activity in the CAD context.

**FIGURE 4:**
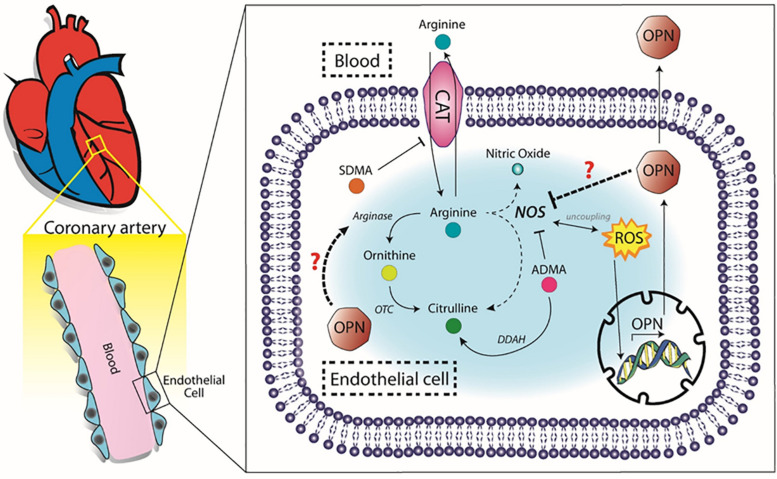
Proposed molecular pathway. OPN, osteopontin, SDMA, symmetric dimethilarginine, ADMA, asymmetric dimetilarginine, CAT, cationic aminoacid transporter, OTC, ornithinetranscarbamilase, DDAH, dimethylarginine dimethylaminohydrolase, ROS, reactive oxygen species.

We strongly believe that OPN could be directly or indirectly implicated in the decreased activity of eNOS in atherosclerosis, contributing to the endothelial dysfunction typically observed in CAD patients. We therefore propose a schematic view of the possible components that could link OPN to the arginine metabolism (Figure 4).

In summary, our results showed a correlation between OPN levels, oxidative stress status, and endothelial dysfunction markers in CAD patients. Nonetheless, further studies are required to determine if OPN really drives the endothelial dysfunction by direct or indirect eNOS inhibition in CAD patients. Endothelial cells from coronary artery, genetically modified to silence or overexpress OPN, could be the appropriate *in vitro* model to determine the functionality of the enzymes involved in the NO/arginine pathway. While OPN knockout mice would represent the best *in vivo* model to evaluate the relationship between the NO/arginine pathway and the OPN (Pedersen et al., 2013).

### Limitations

This study has different limitations. First, we could not investigate the influence of each pharmacological treatment on the analyzed metabolites due to our small cohort. Second, we could not measure eNOS, arginase, and DDAH levels and activity. Third, although flow mediated dilation (FMD) is a recognized technique to assess endothelial dysfunction, we could not evaluate it given the status of our patients before surgery, as well as high number of drugs taken as per the 2019 European Society of Cardiology (ESC) guidelines for FMD evaluation (Thijssen et al., 2019). Lastly, we have not measured other common oxidative stress markers, such as malondialdehyde, since we wanted to investigate the glutathione system and the lipid peroxidation. Our study showed an association between OPN and endothelial dysfunction, however, further studies are necessary to prove the cause-effect relationship in CAD patients.

## Data Availability Statement

The datasets generated for this study are available on request to the corresponding author.

## Ethics Statement

The studies involving human participants were reviewed and approved by the Institutional Review Board and Ethical Committee of Centro Cardiologico Monzino (IRCCS). The patients/participants provided their written informed consent to participate in this study.

## Author Contributions

PP and MD conceived the study. VM collected the informed consensus and the specimens. BP performed mass spectrometry evaluation. VA, VV, and IM performed the experimental evaluations. DM and PP performed statistical analyses and drafted the manuscript. GP prepared the illustration. MD, BP, GP, VV, VA, IM, AO, AD, VC, VM, and PS substantially revised the manuscript. All authors read and approved the final manuscript.

## Conflict of Interest

The authors declare that the research was conducted in the absence of any commercial or financial relationships that could be construed as a potential conflict of interest.
