# Supplementary material for: Relationship Between Plasma Osteopontin and Arginine Pathway Metabolites in Patients With Overt Coronary Artery Disease
Source: Front Physiol. 2020 Aug 6;11:982. doi: 10.3389/fphys.2020.00982 (PMC7424048; doi:10.3389/fphys.2020.00982)
Supplement: Supplementary file 1 [file Data_Sheet_1.docx]

Supplementary Material

**Supplementary Table S1.**

Cohort’s demographics and clinical characteristics.

| **Variables** | **CAD patients**  **(n = 33)** | **CTRL group**  **(n = 25)** | **P value** |
| --- | --- | --- | --- |
| Sex (male) n, (%) | 32 (97) | 17 (68) | **0.004** |
| Age, years | 63.0 ± 8.1 | 58.6 ± 10.9 | **0.02** |
| Diabetes n, (%) | 0 (0) | 0 (0) | 1.00 |
| Hypertension n, (%) | 26 (78,8) | 11 (44) | **0.01** |
| Dyslipidemia n, (%) | 25 (75,8) | 12 (48) | 0.05 |
| Smoking habits n, (%) | 8 (24,2) | 4 (16) | 0.53 |
| BMI, kg/m^2^ | 27.6 ± 3.5 | 26.8 ± 3.7 | 0.52 |
| e-GFR, mL/min/1.73m^2^ | 89.2 ± 16.2 | 98.8 ± 19.8 | **0.02** |
| LVEF | 59.2 ± 10.5 | - | - |
| ***Pharmacological Therapies*** | | | |
| ACE-inhibitors n, (%) | 8 (24,2) | 5 (20) | 0.76 |
| Antiplatelets n, (%) | 21 (63,6) | 1 (4) | **< 0.0001** |
| β-blockers n, (%) | 24 (72,7) | 1 (4) | **< 0.0001** |
| Ca-antagonists n, (%) | 7 (21,2) | 2 (8) | 0.27 |
| Nitrates n, (%) | 11 (33,3) | 0 (0) | **0.001** |
| Statins n, (%) | 19 (57,6) | 6 (24) | **0.01** |

CAD: coronary artery disease; CTRL: control; BMI: body mass index; e-GFR: estimated glomerular filtration rate; LVEF: left ventricular ejection fraction. Data are shown as mean ± standard deviation or frequence (n) and percentage (%).

**Supplementary Figure S1.**

Circulating osteopontin (OPN) levels in controls (CTRL) and coronary artery disease (CAD) patients. *p < 0.05.


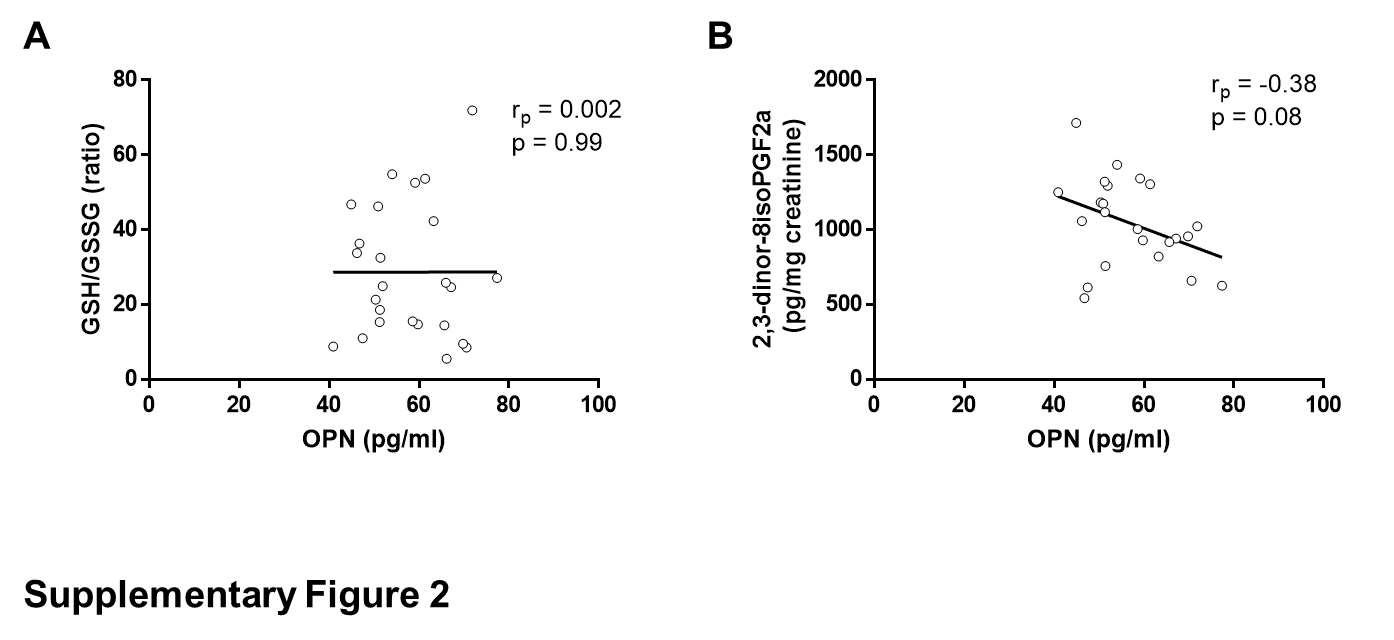


**Supplementary Figure S2.**

Linear regression analyses between osteopontin (OPN) and GSH/GSSG ratio (**A**) and 2,3-dinor-8isoPGF2a (**B**) in control group.


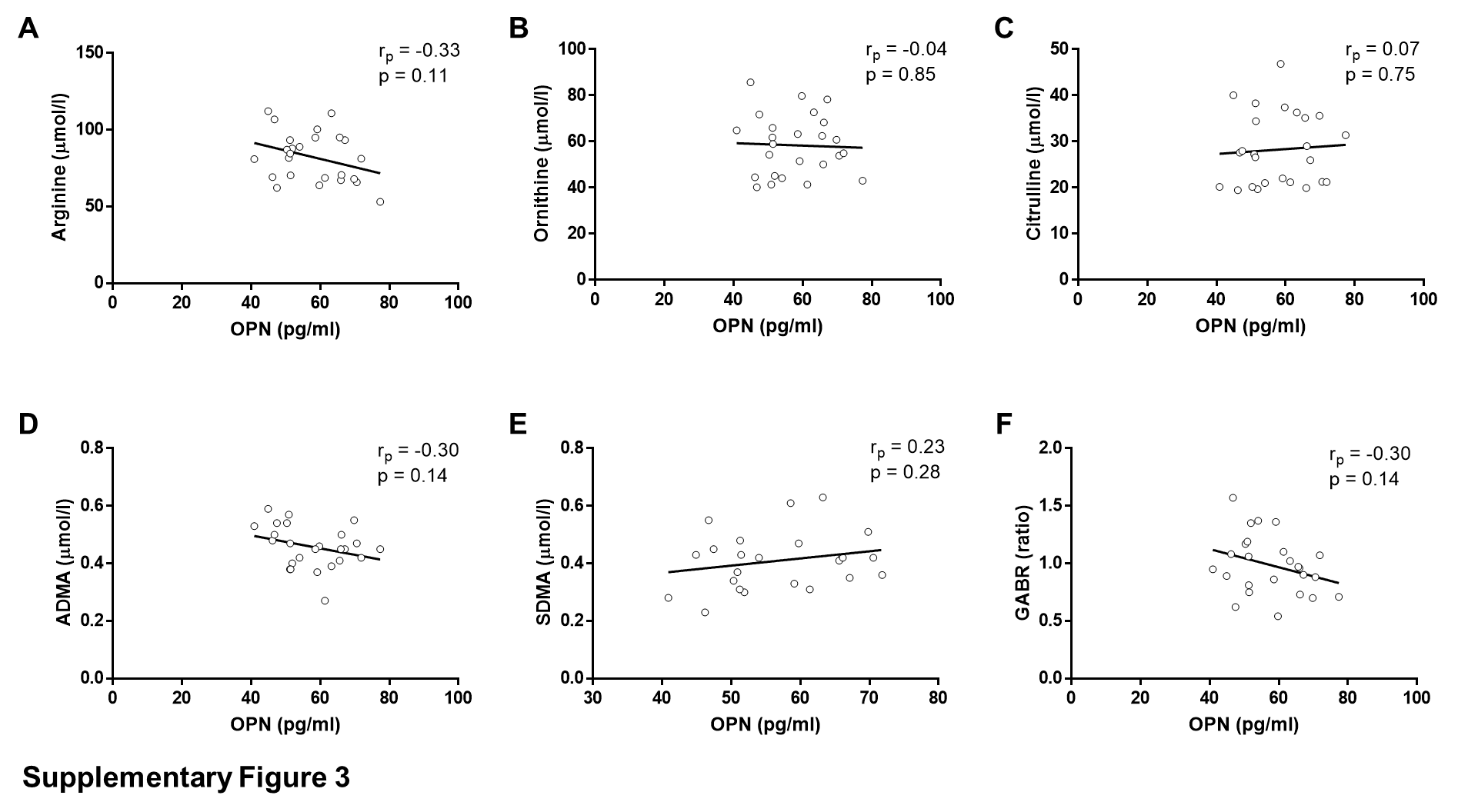


**Supplementary Figure S3.**

Linear regression analyses between osteopontin (OPN) and arginine (**A**), ornithine (**B**), Citrulline (**C**), asymmetric dimetilarginine (ADMA, **D**), symmetric dimethilarginine (SDMA, **E**), and global arginine bioavailability (GABR, **F**) in control group.
